# Genome-Wide Identification, Characterization and Expression Analysis of the Solute Carrier 6 Gene Family in Silkworm (*Bombyx mori*)

**DOI:** 10.3390/ijms17101675

**Published:** 2016-10-03

**Authors:** Xin Tang, Huawei Liu, Quanmei Chen, Xin Wang, Ying Xiong, Ping Zhao

**Affiliations:** 1 State Key Laboratory of Silkworm Genome Biology, Southwest University, Chongqing 400715, China; 13098784616@163.com (X.T.); lhw888718@163.com (H.L.); wangxin1-4@163.com (X.W.); xiongy0823@126.com (Y.X.); 2 Department of Biochemistry and Molecular Biology, Chongqing Medical University, Chongqing 400016, China; eidoloncqm@126.com; 3 Chongqing Engineering and Technology Research Center for Novel Silk Materials, Chongqing 400715, China

**Keywords:** *Bombyx mori*, expression pattern, motor behavior, nutrient absorption, the solute carrier 6 (SLC6) gene

## Abstract

The solute carrier 6 (SLC6) gene family, initially known as the neurotransmitter transporters, plays vital roles in the regulation of neurotransmitter signaling, nutrient absorption and motor behavior. In this study, a total of 16 candidate genes were identified as SLC6 family gene homologs in the silkworm (*Bombyx mori*) genome. Spatio-temporal expression patterns of silkworm SLC6 gene transcripts indicated that these genes were highly and specifically expressed in midgut, brain and gonads; moreover, these genes were expressed primarily at the feeding stage or adult stage. Levels of expression for most midgut-specific and midgut-enriched gene transcripts were down-regulated after starvation but up-regulated after re-feeding. In addition, we observed that expression levels of these genes except for *BmSLC6-15* and *BmGT1* were markedly up-regulated by a juvenile hormone analog. Moreover, brain-enriched genes showed differential expression patterns during wandering and mating processes, suggesting that these genes may be involved in modulating wandering and mating behaviors. Our results improve our understanding of the expression patterns and potential physiological functions of the SLC6 gene family, and provide valuable information for the comprehensive functional analysis of the SLC6 gene family.

## 1. Introduction

The solute carrier 6 (SLC6) gene family of membrane transporters shuttle extracellular neurotransmitters, amino acids and osmolytes across the plasma membrane into the cytoplasm through co-transport of Na^+^ and Cl^−^. The Na^+^ electrochemical potential difference is the driving force used to transport substrates. In insects, some SLC6 members also depend on the existence of K^+^ [1,2,3,4]. Generally, the SLC6 gene family, also referred to as the sodium neurotransmitter symporter family (SNF), includes an SNF domain, the structural fingerprints of SLC6 transporters [5,6]. SLC6 transporters have approximately 12 highly-conserved transmembrane (TM) domains, most notably TM1, TM6 and TM8. In addition, they contain a functional core of 10 TM helices (TMs): an inverted-topological repeat, TMs1-5 and TMs6-10 [7,8]. These TM domains contain the binding sites for substrates and ions [7].

The SLC6 family is currently divided into four subfamilies that are monoamine transporters, γ-aminobutyric acid (GABA) transporters, amino acid transporters (I), and amino acid transporters (II)/orphan transporters [9]. Monoamine transporters, GABA transporters, and glycine transporters are referred to as neurotransmitter transporters (NTTs). NTTs play prominent roles in maintaining physiological homeostasis and regulating neurotransmitter signaling through the uptake or release of a neurotransmitter in neurons and glial cells [10]. However, the amino acid transporters (II) subfamily, which accepts a broad spectrum of substrates, differs from the other aforementioned transporters in physiological function. This particular subfamily is involved in the neutral amino acid absorption and play important roles in various metabolic processes [11,12,13]. Unlike mammals, the SLC6 gene family in insects is commonly separated into five branches, including neurotransmitter transporters (NTTs), amino acid transporters (AATs), nutrient amino acid transporters (NATs), INEs (inebriated genes), and orphan transporters [12]. Moreover, evolution of genes in the SLC6 family differs between vertebrates and invertebrates. For example, the insect-specific nutrient amino acid transporter (iNAT) cluster transports the essential and neutral amino acids into the cell [5,14]. While iNATs are functionally similar to mammalian amino acid transporters (II), they are classified into a single SLC6 subfamily in insects.

Neurotransmitters are important in the regulation of homeostasis and biological processes. Thus, many earlier studies have focused on NTTs, and their functions have been demonstrated with abundant evidence. For example, a dopamine transporter mediates dopamine homeostasis and is related with schizophrenia, Parkinson’s disease, and ADHD [15,16]. In addition, a GABA transporter controls GABA homeostasis and plays an important role in anxiety disorders [17]. These results suggested that NTTs are involved in various physiological processes, such as locomotor activity, anxiety behaviors, and stress behaviors [18,19,20,21]. Recently, X-ray crystallographic structures of the dopamine transporter of *Drosophila melanogaster* and the serotonin transporter of humans were reported, which provide blueprints for future drug design [8,22]. To accurately describe the SLC6 gene family, the physiological functions of additional subfamily genes were also studied. Unlike NTTs, insect NATs demonstrate the ability to absorb essential and neutral amino acids in alimentary canal. Tissue localization of *D. melanogaster* SLC6 transporters through RT-PCR and in situ hybridization [23], further promotes the study of insects SLC6 members in insects. Subsequently, several insect NATs have been identified and characterized. For instance, AgNAT6 and AgNAT8, SLC6 members in the *Anopheles gambiae*, transport phenol-branched and indole-branched substrates [5,24]; MsCAATCH1 and MsKAAT1 from the *Manduca sexta* have been shown to have narrow substrates spectrum and are responsible for proline and threonine transport [25]; and DmNAT1, the first cloned NAT from *Drosophila* mediates the absorption of d-amino acids [26].

Compared with the studies of other insect SLC6 transporters, there is little knowledge about SLC6 family genes in the silkworm, *Bombyx mori*. To investigate the role of SLC6 family genes in silkworm, bioinformatic analyses were performed to identify SLC6 transporters homologs in the silkworm genome. Moreover, phylogenetic relationships among insect SLC6 members and spatio-temporal expression profiles of silkworm SLC6 family members were determined. In addition, starvation experiments and juvenile hormone analog (JHA) treatment were performed to functionally assess midgut-enriched and midgut-specific SLC6 genes. Together, our results will further enhance studies of the evolution and function of SLC6 genes in insects.

## 2. Results

### 2.1. Identification of the Solute Carrier 6 (SLC6) Transporter Homologs in the Silkworm Genome

To identify all SLC6 transporter homologs in the silkworm genome, we performed a BLASTP search against the NCBI GenBank and the Silkworm Genome Database (SilkDB) using known SLC6 protein sequences from other species. Subsequently, a total of 16 transporters were identified in the silkworm genome, including two previously identified SLC6 genes, namely *BmDAT* and *BmSERT* (Table 1). These silkworm transporters include the SNF domain that is a typical of SLC6 transporters. These silkworm SLC6 transporter proteins ranged from 538 to 727 amino acids (with the exception of BmSLC6-16). SLC6 family genes were found to be broadly distributed across the silkworm chromosomes, and the total number of silkworm SLC6 genes was similar to numbers reported in *D. melanogaster*, *A. gambiae*, and *A. aegypti*. Silkworm SLC6 genes were named according to the annotations, information from NCBI and SilkDB, and previous studies [27,28].

### 2.2. Multiple Sequence Alignment of the B. mori SLC6 Transporters

**Figure 1:**
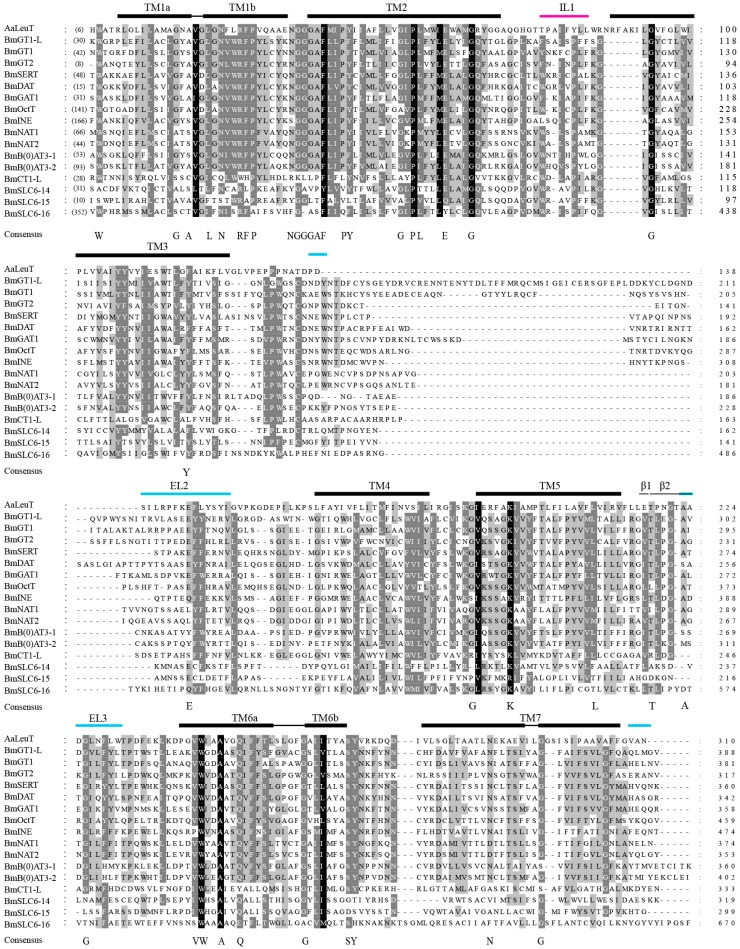

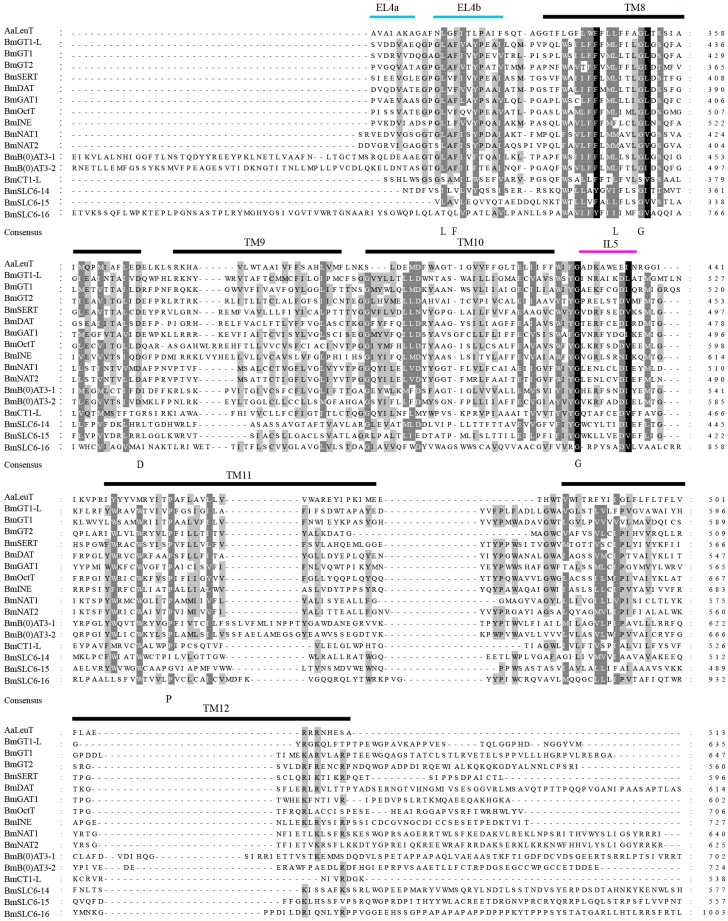
A multiple sequence alignment (MSA) of *Bombyx mori* the Solute Carrier 6 (SLC6) proteins with *A. aeolicus* LeuT_Aa_. The protein domains, including transmembrane (TM) regions, extracellular linker (EL) regions (blue lines), intracellular linker (IL) regions (pink lines), alpha helical structure (thick black line), and beta sheets (tiny black lines), are annotated at the top of each sequence block. The annotation of protein domains is based on published studies [7,23]. The consensus is absolutely conserved for amino acid residues in the sequence alignment of Yamashita [7]. The background of amino acid residues is based on the degree of conservation (black = 100%, dark gray = 80%, light gray = 60%).

The reported crystal structure of *Aquifex aeolicus* LeuT_Aa_ reveals important residues for substrate binding and ion binding [7]. As a result, we chose LeuT_Aa_ as a control. Amino acid sequence alignments of *B. mori* SLC6 transporters with *A*. *aeolicus* LeuT_Aa_ are presented in Figure 1. Among all silkworm SLC6 members, 10 to 13 predicted TM domains were identified in the multiple sequence alignment (MSA) (data not shown). The MSA revealed that *B. mori* SLC6 transporters contain an inverted structural repeat of transmembrane helices TMs1-5 and TMs6-10 (Figure 1). Multiple residues were found to be highly conserved in TM1, TM2, TM6, and TM8. However, a few invariant residues were identified in TM2 and TM6, including glycine, proline, tryptophan, and alanine, which differ from invariant residues reported in other studies [7,23]. Furthermore, the NTTs of *B. mori* (BmSERT, BmDAT, BmGAT1, BmOctT, BmGT1, and BmGT2) showed 45% sequence identity.

### 2.3. Phylogenetic Analysis of the Insect SLC6 Family Members

**Figure 2:**
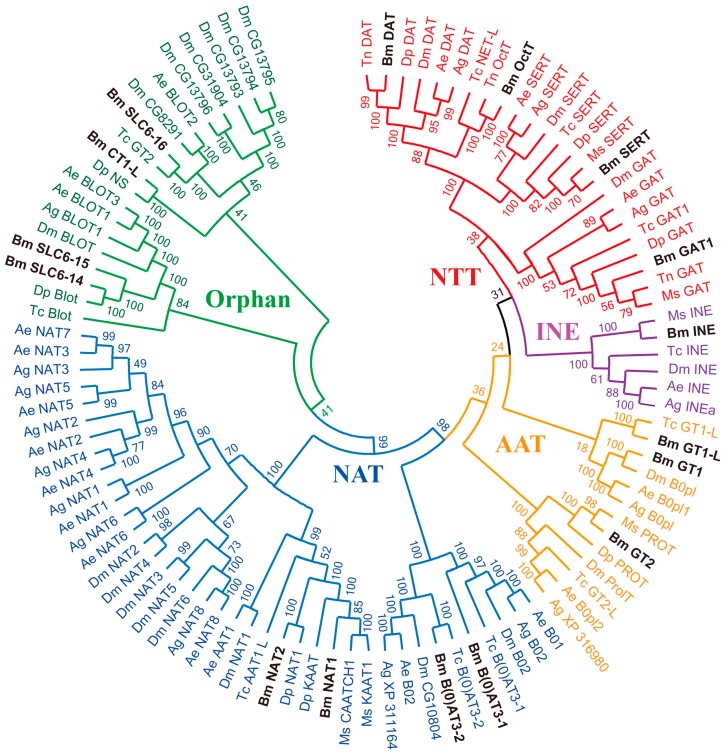
Phylogenetic tree of SLC6 transporters from *B. mori* and other insect species. Colors represent different subfamilies or branches. NTT: neurotransmitter transporter, AAT: amino acid transporter, INE: inebriated gene, NAT: nutrient amino acid transporter, Orphan: orphan transporter. SLC6 transporters from *D. melanogaster* (Dm)*, A. gambiae* (Ag), *and A. aegypti* (Ae), whose genome sequences have been reported [29,30,31], were selected to characterize the evolutionary relationships of insect SLC6 transporters. *B. mori* SLC6 transporters are highlighted in boldface.

To further elucidate the phylogenetic relationship among insect SLC6 transporters, the silkworm SLC6 genes were compared to known insect SLC6 proteins using a phylogenetic approach. A total of 101 insect SLC6 sequences were used to construct the phylogenetic tree. The phylogenetic tree includes 16 SLC6 proteins from *B. mori* and 85 SLC6 proteins from other insects: 21 from *D. melanogaster*, 20 from *A. aegypti*, 16 from *A. gambiae*, 11 from *Tribolium castaneum* (*T. castaneum)*, eight from *Danaus plexippus* (*D. plexippus)*, six from *M. sexta*, and three from *Trichoplusia ni* (*T. ni* *)* (Figure 2). All amino acid sequences are listed in the Supplementary File: Table S1. Indeed, the SLC6 phylogenetic tree clustered into five branches: NTT, INE, AAT, NAT, and Orphan (Figure 2), similar to previous studies [5,12]. Four transporters (BmDAT, BmGAT1, BmSERT, and BmOctT) were classified into the known NTT subfamily that regulates neurotransmitter signals. In addition, four transporters (BmSLC6-14, BmSLC6-15, BmSLC6-16, and BmCT1-L) were found to belong to the Orphan subfamily, whose function is still unknown in insects.

### 2.4. Spatial and Temporal Expression Profiles of the Silkworm SLC6 Genes

**Figure 3:**
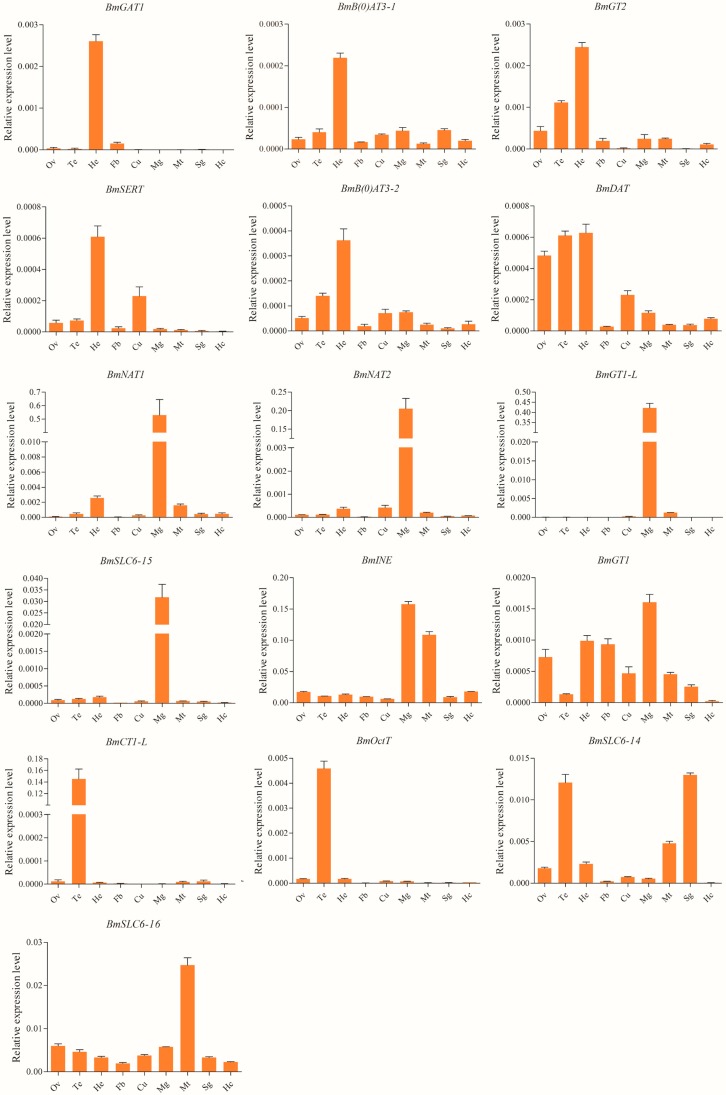
The tissue expression profiles of silkworm SLC6 family genes on third day of the fifth instar. The cDNA templates were derived from ovaries (Ov), testis (Te), head (He), cuticles (Cu), fat body (Fb), midgut (Mg), hemocytes (Hc), Malpighian tubules (Mt), and silk gland (Sg).

Tissue expression profiles of silkworm SLC6 genes in nine tissues collected from third day of the fifth instar larvae were performed using quantitative real-time PCR (Figure 3). *BmNAT1*, *BmNAT2*, *BmGT1-L*, and *BmSLC6-15* were specifically expressed in the silkworm midgut. The level of *BmINE* mRNA expression was high in midgut and Malpighian tubules, but lower in other tissues. Moreover, *BmGT1* was expressed in all tissues except for the hemocytes. Compared to other tissues, *BmGT1* showed high levels of mRNA expression in midgut. Six genes (*BmGAT1*, *BmGT2*, *BmDAT*, *BmSERT*, *BmB(0)AT3-1*, and *BmB(0)AT3-2*) were highly expressed in the heads of silkworms, particular *BmGAT1* and *BmGT2*. Subsequently, we further found that the aforementioned six genes are highly expressed in silkworm brain. *BmDAT*, the dopamine transporter gene, is also expressed at high levels in gonads. In addition, *BmSERT*, the serotonin transporter gene, is also expressed in the cuticle. Similarities were found in the expression patterns of *BmGT2* and *BmB(0)AT3-2*, which were found to be expressed in the ovaries and testis. *BmCT1-L* and *BmOctT* were specifically expressed in testis. The level of *BmSLC6-14* mRNA expression was high in testis and the silk gland, but low in the ovaries, head, and Malpighian tubules. Furthermore, *BmSLC6-16* was found to be highly expressed in Malpighian tubules and less so in other tissues.

**Figure 4:**
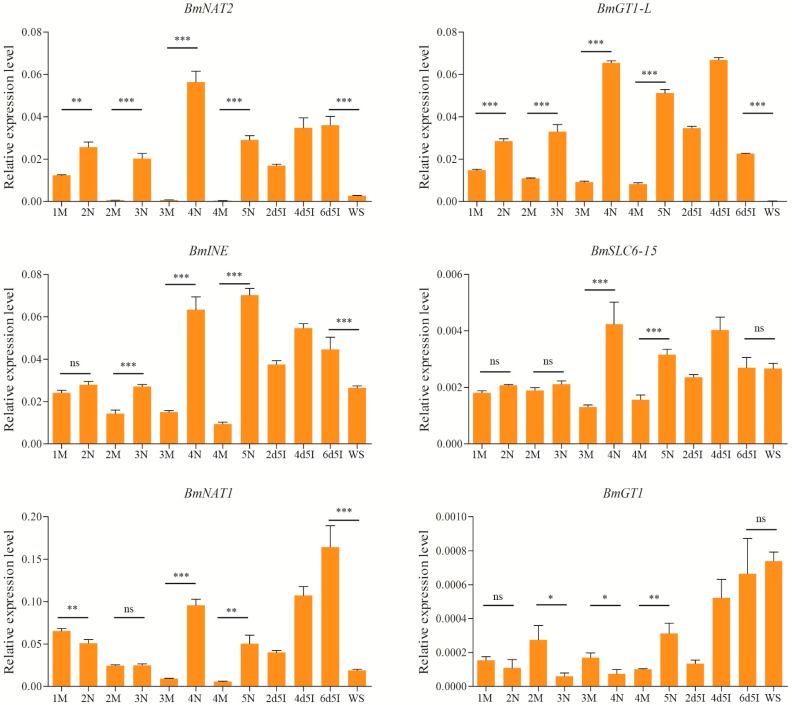
Analysis of temporal expression patterns of midgut-specific and midgut-enriched SLC6 members. M: molting larvae; N: newly molted larvae; 2d5I, 4d5I, and 6d5I: Days 2, 4, and 6 of the fifth instar, respectively; WS: wandering stage. * *p* < 0.05, ** *p* < 0.01 and *** *p* < 0.001. ns: no significant differences (*p* > 0.05).

The midgut is major tissues involved in nutrient digestion and absorption in insects. Thus, midgut-specific and midgut-enriched silkworm SLC6 genes were selected to detect temporal expression patterns in the larval stage. Expression levels of *BmINE*, *BmNAT2*, *BmGT1-L*, and *BmSLC6-15* in newly molted larvae were found to be higher than in molting larvae, and there was a significantly difference between newly molted larvae and molting larvae (Figure 4). Moreover, these genes showed persistent expression in the fifth instar. Compared to Day 6 of the fifth instar, levels of *BmNAT2*, *BmGT1-L*, and *BmNAT1* mRNA were low in the wandering stage, and *BmINE* displayed the same expression pattern. These genes were significantly down-regulated in the wandering stage. It is noteworthy that the temporal expression profiles of *BmGT1* were different from all others genes assessed. Indeed, within the first, second, third, and fourth larval instars, the mRNA levels of *BmGT1* were higher in molting larvae than in newly molted larvae.

### 2.5. Expression Levels of Brain-Enriched Genes in Wandering and Mating Behaviors

**Figure 5:**
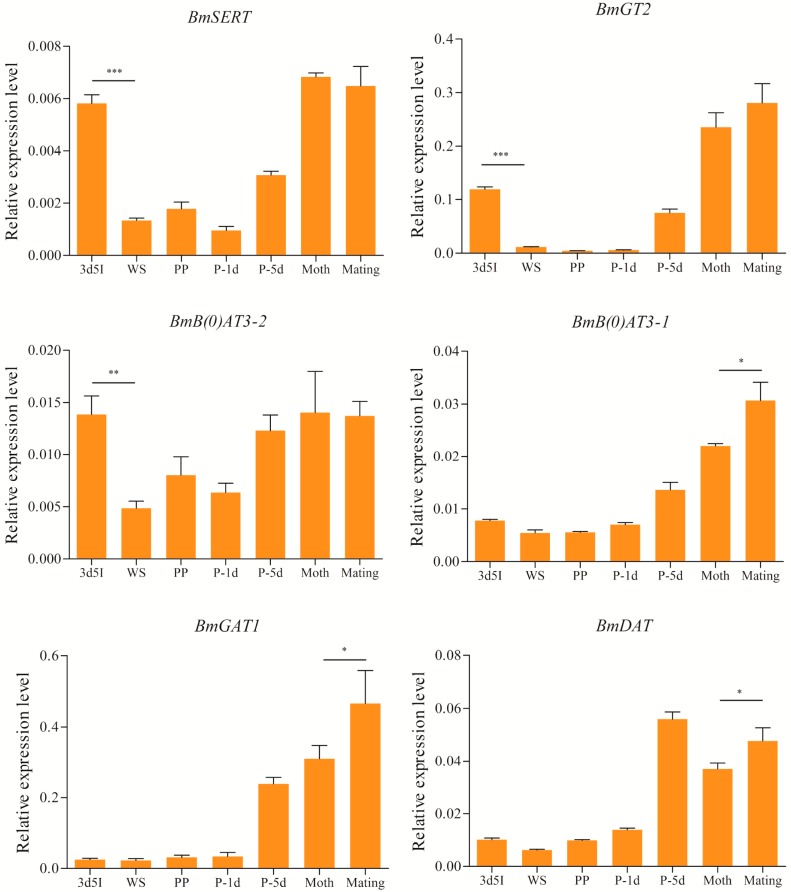
Expression levels of brain-enriched genes in mating and wandering behavior. Error bars indicate the standard error of the mean (*n* = 3). 3d5I: feeding stage; PP: before pupation; P-1d: the first day after pupation; P-5d: the fifth day after pupation; Moth: newly molted moth. * *p* < 0.05, ** *p* < 0.01 and *** *p* < 0.001.

As a result of increased feeding for the last instar larvae and the behavior changes during the wandering and adult stages, we further investigated mRNA expression patterns of head-enriched SLC6 genes in silkworm brain during larval-pupal-adult development. The cDNA templates were derived from the silkworm brains in this experiment and levels of gene expression were examined in dissected brains. Levels of mRNA transcripts were used to screen for differential expression of genes related to wandering and mating behaviors (Figure 5). As a result, *BmSERT*, *BmGT2*, and *BmB(0)AT3-2* were found to show differential expression between the feeding and wandering stages. The third day of the fifth instar (3d5I), the boundary of larval development [32], was selected to represent the feeding stage. These genes were found to be highly expressed at the feeding stage, but mRNA transcript levels of these genes were low at the wandering stage. However, other genes (*BmGAT1*, *BmDAT*, and *BmB(0)AT3-1*) showed an identical trend during the feeding and wandering stages. Compared to other developmental stages, these genes were highly expressed at the middle pupal and adult stages. Brain-enriched genes, except for *BmB(0)AT3-2* and *BmDAT*, were expressed at lower levels in the early pupal stage. In summary, these findings serve as further validation that head-enriched SLC6 genes are highly expressed in brain tissue. Together, these results imply that brain-enriched genes may be involved in wandering and mating behaviors.

### 2.6. The Influences of Starvation on the Expression of the Silkworm SLC6 Genes

**Figure 6:**
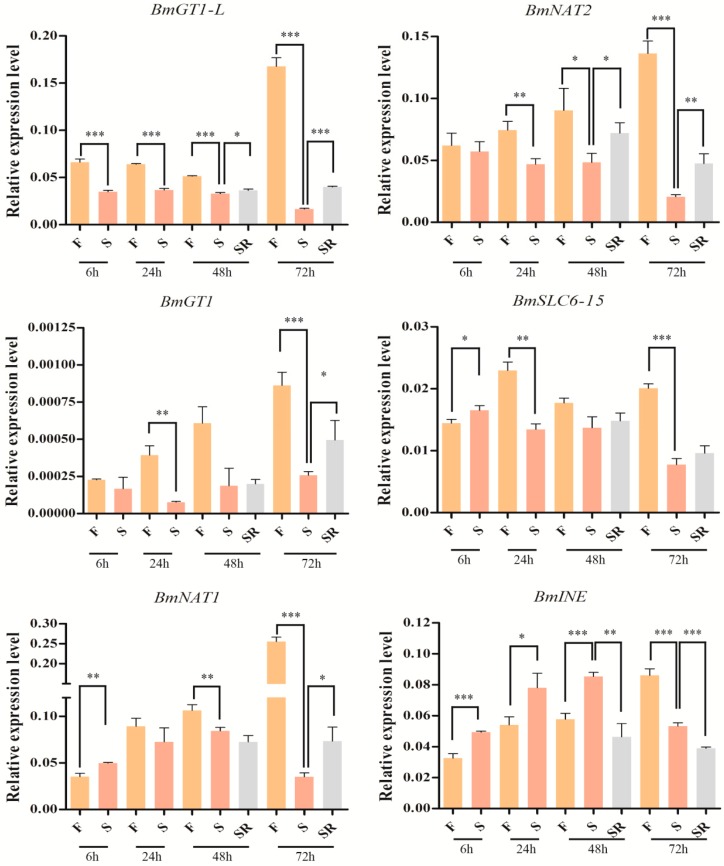
Influences of starvation on the expression of midgut-specific and midgut-enriched SLC6 genes. F: feeding; S: starvation; SR: starvation and re-feeding. Significant differences were assessed by a student’s *t*-test (* *p* < 0.05, ** *p* < 0.01 and *** *p* < 0.001). Error bars indicate the standard error of the mean (*n* = 3).

Genes in the SLC6 family were found to be highly and specifically expressed in the alimentary canal, mediating nutrient absorption, such as essential and neutral amino acids or amino acid-like substrates [12,33]. Therefore, taking into account the spatio-temporal expression pattern of silkworm SLC6 family transporters, midgut-enriched and midgut-specific genes were selected to determine their associations with starvation and re-feeding (Figure 6). *BmGT1-L*, *BmNAT2* and *BmGT1* were significantly down-regulated by starvation and up-regulated again by re-feeding. In contrast, with a starvation time of 6 h, *BmNAT1* and *BmSLC6-15* were up-regulated; afterwards, these genes were down-regulated by starvation. However, *BmINE* was up-regulated by starvation in most of treatments and down-regulated by re-feeding. These results indicated that midgut-enriched and midgut-specific SLC6 genes may be closely related to silkworm nutrient absorption.

### 2.7. Induced Expression of SLC6 Genes by Juvenile Hormone Analog (JHA)

**Figure 7:**
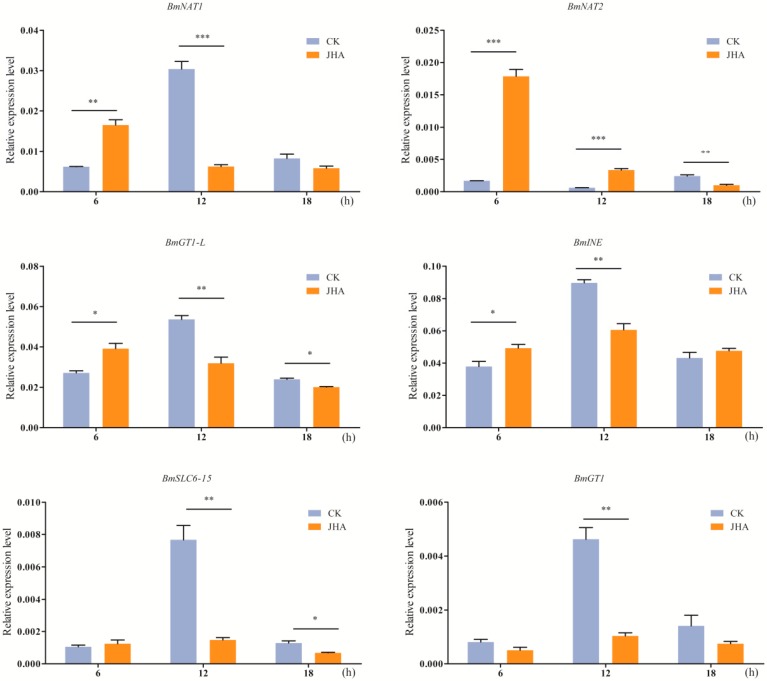
Induced expression of midgut-specific and midgut-enriched SLC6 genes by juvenile hormone analog (JHA) in ex-vivo midgut. Significant differences were assessed by a student’s *t*-test (* *p* < 0.05, ** *p* < 0.01 and *** *p* < 0.001). Error bars indicate the standard error of the mean (*n* = 3). CK: control group; JHA: juvenile hormone analog.

We found that some SLC6 genes were highly expressed after molting (Figure 4). Similarly, it has been reported that a peak of JH I (juvenile hormone I) is observed after ecdysis in the silkworm larval stage, and the JH I titer reaches the lowest level before ecdysis [34]. Therefore, we hypothesized that the genes were correlated with circulating JH I. To determine whether JH induces SLC6 gene expression, the midguts, which derive from the fourth molting larvae, were separated from larval bodies and incubated with JHA (1 μM). Results from qPCR showed that expression levels of *BmNAT1*, *BmNAT2*, *BmGT1-L*, and *BmINE* were significantly induced after incubation with JHA for 6 h (Figure 7). However, *BmGT1* and *BmSLC6-15* showed no difference after JHA treatment for 6 h, but they were markedly down-regulated by JHA treatment for 12 h (Figure 7).

## 3. Discussion

The SLC6 gene family plays an important role in various physiological processes, including motor behavior, nutrient absorption, and transmission of neurotransmitter signals [10,33]. Previous studies have reported 21 SLC6 genes in *D. melanogaster*, 20 in *A. aegypti*, and 17 in *A. gambiae* [12,23]. In this study, 16 SLC6 genes were identified in *B. mori* (Table 1). Moreover, their structural features, phylogenetic relationships, and expression patterns were elucidated.

It is well known that protein function is largely predicated on structure. The structural description of silkworm SLC6 proteins can contribute to understanding their potential functions. It is noteworthy that a major difference in sequence alignment between our results and those reported by Yamashita [7] is the inclusion of additional sequences. In this study, four transporters (BmOctT, BmINE, BmSLC6-16 and BmB(0)AT3-2) were predicted to have large intracellular *N*-termini that may be modified by protein–protein interactions or phosphorylation [35]. The extracellular loop and TM domain may mediate substrate binding and inhibitor binding [36]. However, three proteins (BmB(0)AT3-1, BmB(0)AT3-2, and BmSLC6-16) were predicted to have a long extracellular loop between TM7 and TM8. Moreover, BmGT1-L was predicted to have a large loop between TM3 and TM4. Therefore, for these proteins, substrate binding may be affected by the addition of an extracellular loop. A previous study has demonstrated that TM1 and TM6 play key roles in substrate binding and transport, and TM3 and TM8 are also involved in substrate transport [7]. Interestingly, TM1 and TM6 in silkworm SLC6 proteins (BmCT1-L, BmSLC6-14, BmSLC6-15, and BmSLC6-16) were more divergent (Figure 1), which is similar to published findings [23]. Accordingly, we infer that capacity for substrate binding in these proteins may be different from other members.

Phylogenetic analyses inferred the classification of insect SLC6 genes into five major classes: NTT, INE, AAT, NAT, and Orphan (Figure 2). Firstly, the NTT includes four transporters: GABA transporters, dopamine transporters, 5-HT transporters and octopamine transporters, which are mainly expressed in brain and are involved in regulating insect behavior [37,38,39,40]. In *B. mori*, the NTT genes were also found to be highly expressed in brain, implying that NTT may participate in similar physiological processes. Based on spatio-temporal expression patterns of silkworm genes in the SLC6 family, we found that expression levels of *BmGAT1* and *BmGT2* are much higher than other brain-enriched genes. It has been speculated that *BmGAT1* and *BmGT2* may be more important for behavior regulation than other genes in the brain; Secondly, the INE is encoded by the *inebriated* gene, which is closely related to motor neuron excitability in *D. melanogaster* [41,42], and INE is also essential for the regulation of the systemic water homeostasis in *Drosophila* hindgut [43]. Interestingly, *BmINE* was highly expressed in midgut and Malpighian tubules, indicating that the function of INE in silkworm may be different from that in *D. melanogaster*; additionally, the AAT mainly includes glycine and proline transporters, which are responsible for regulating glycine and proline levels in glycinergic and glutamatergic synapses, respectively. [33,44,45]. Silkworm glycine transporters (*BmGT1*, *BmGT1-L* and *BmGT2*) were found to be highly expressed in brain and midgut, indicating that the glycine transporters may differ functionally between *B. mori* and *D. melanogaster*; Finally, the NAT includes numerous neutral amino acid transporters and a few B^0+^ transporters, which are expressed in the alimentary canal and mediate nutrient absorption [46]. In this study, four silkworm SLC6 genes (*BmNAT1*, *BmNAT2*, *BmB(0)AT3-1*, and *BmB(0)AT3-2*) were classified into the NAT subfamily. Among midgut-specific and midgut-enriched genes, expression levels of *BmGT1-L* and *BmNAT1* were higher than the others tested. This result implies that these genes may play important roles in nutrient absorption.

Wandering behavior is important for pupation [47] and mating behavior is essential to reproduction and survival in insects [48,49], so it is crucial to analyze the molecular basis of these behaviors. There is a vast difference in motor behaviors between the feeding and wandering stages [40]. During the early wandering stage, the silkworm cuticula is transparent and the larva shows enhanced locomotor activity [40]. Additionally, it is well known that the brain is the central organ of central nervous system (CNS), and controls several motor behaviors [50]. Therefore, we investigated the expression levels of brain-enriched SLC6 genes during wandering and mating processes. The results show that *BmGT2* and *BmSERT mRNA* expression levels are very low at the wandering stage (Figure 5). Interestingly, glycine is the main inhibitory neurotransmitters in the CNS, and the concentration of glycine is regulated by glycine transporters (GlyTs) [51]. Thus, the expression pattern of *BmGT2* implies that glycine transport in the brain may be reduced at the wandering stage. Compared to the newly molted moth stage, *BmB(0)AT3-1*, *BmDAT*, and *BmGAT* were highly expressed during mating, which implies that these genes may be involved in mating behavior and reproduction. Moreover, it is noteworthy that the expression level of *BmGAT* is greatly higher than *BmB(0)AT3-1* and *BmDAT*, implying that *BmGAT* may play an important roles in the genetic basis of mating behavior.

There were drastic changes in silkworm tissues and metabolic processes after starvation, for example, the process of DNA synthesis in the silk gland cell can be markedly down-regulated by starvation and re-activated by re-feeding [52]. In addition, starvation rapidly up-regulated the mRNA levels of *InR*, *IRS*, *PI3K110*, and *PDK* in the fat body. These genes play key roles in the insulin/insulin growth factor signaling (IIS) pathway that regulates nutrition-dependent growth rates of insects [53]. These results imply that the genes involved in nutrition metabolism and growth may be affected by starvation or re-feeding. In this study, three genes (*BmGT1-L, BmGT1* and *BmNAT2*) were induced by starvation or re-feeding (Figure 6). Therefore, we speculate that these genes may be related to nutrition metabolism. However, more evidence and trials are needed to clarify the function of these genes. Curiously, the expression of *BmINE* was up-regulated by starvation. Thus, we suggested that *BmINE* may be involved in other physiological processes. For instance, *INE* plays a key role in regulating systemic water homeostasis in Drosophila hindgut [43].

In insects, JH plays an important role in many physiological processes, including development, reproduction, and metamorphosis [34]. In some insect species, SLC6 family NAT genes are induced by JH. For example, the *D. melanogaster JhI-21* and *minidiscs* (*mnd*) are JH-inducible [54]. In *Leptinotarsa decemlineata*, JH and a JH analog can activate *LdNAT1* expression [55]. In this study, the expressions of four genes (*BmNAT1*, *BmNAT2*, *BmGT1-L*, and *BmINE*) were up-regulated by JHA (Figure 7). These results suggest that most of midgut-enriched and midgut-specific SLC6 genes are induced by JHA. Furthermore, they imply that JH may be the upstream regulator of certain SLC6 genes.

In conclusion, our study provides a comprehensive analysis of the SLC6 family in the silkworm, including gene conservation, evolution, expression patterns, and potential functions. Together, these results will promote functional studies of insect SLC6 genes, and improve our understanding of insect ethology.

## 4. Materials and Methods

### 4.1. Insect Rearing and Sample Collection

The silkworm strain (Dazao) was provided by the State Key Laboratory of Silkworm Genome Biology, Southwest University, Chongqing, China. The larvae were routinely reared on fresh mulberry leaves at 25 ± 1 °C and humidity of 75% ± 5% under 14 h light/10 h dark cycles. For tissue expression analysis, different tissue samples from the third day of the fifth instar larvae were dissected on ice. For temporal expression analysis, samples were collected at various instars. Each sample contained at least 3 larvae. The larval brains were collected at different time point that is related to wandering behavior and mating behavior. All samples were stored at −80 °C for RNA isolation.

### 4.2. Identification and Multiple Sequence Alignment of the Silkworm SLC6 Family Transporters

BLASTP searches of predicted proteins were performed against NCBI and the Silkworm Genome Database (http://www.silkdb.org/silkdb/) to identify the candidate genes of the silkworm SLC6 family. Monoamine transporters, GABA transporters, orphan transporters, and Nutrient Amino acid transporters from other species were used for sequence searches. Subsequently, the SMART (http://smart.embl-heidelberg.de/) and Pfam (http://pfam.sanger.ac.uk/) databases were used to validate each candidate transporter. Candidate transporters, which have the SNF domain and high sequence identity, were considered as SLC6 gene family members. The sequences of SLC6 transporters from *D. melanogaster*, *A. gambiae*, *A. aegypti*, *M. sexta*, *T. ni*, *D. plexippus* and *T. castaneum* were downloaded from the NCBI GenBank, VectorBase and FlyBase. A multiple sequence alignment, including 16 putative SLC6 genes from *B. mori* and the leucine transporter from *A. aeolicus* [7], was constructed using the ClustlX version 1.8. The identified protein sequences were aligned according to the previously published studies [23]. Finally, the alignment was artificially edited and adjusted using GeneDoc software (www.psc.edu/biomed/genedoc).

### 4.3. Phylogenetic Tree Construction

Phylogenetic analyses were performed using MEGA version 5.02 (http://www.megasoftware.net). The phylogeny was constructed according to the Poisson correlation model generated by the neighbor-joining (NJ) method, and the bootstrap value was set to 1000. To describe evolutionary relationships among insect SLC6 members, the alignment of 101 SLC6 family transporters was selected. Homologs protein sequences were derived from Coleopteran, Lepidoptera, and Diptera, including *D. melanogaster* and *B. mori*.

### 4.4. Quantitative Real-Time PCR (qPCR) Analysis

Total RNA from various samples was isolated using the EZNA Total RNA Kit (Omega Bio-Tek Inc., Norcross, GA, USA) according to the manufacturer’s instructions. Then, DNaseI (Invitrogen™, Thermo Fisher Scientific, Waltham, MA, USA) was used to remove residual DNA. Four micrograms of total RNA were used to synthesize first-strand of cDNA with M-MLV Reverse Transcriptase (Invitrogen™, Thermo Fisher Scientific, Waltham, MA, USA) at 42 °C. Silkworm SLC6 genes mRNA sequences were obtained from the NCBI GenBank sequence database. Forward and reverse primers for silkworm SLC6 genes were designed using the Primer Premier version 5.0 (Premier Biosoft International, Palo Alto, CA, USA). Eukaryotic translation initiation factor 4A (*BmeIF-4a*, SilkDB Probe: sw22934) of *B. mori* was used as an internal control. The oligonucleotide primers corresponding to silkworm SLC6 family genes used in this study are listed in Supplementary File: Table S2.

The 7500 Fast Real Time PCR System (Applied Biosystems, Foster City, CA, USA) and a SYBR^®^ Premix Ex Taq™ Kit (TaKaRa BIO Inc., Otsu, Japan) were used according to the manufacturer’s instructions for qPCR. The qPCR was performed under the following conditions: initial denaturation for 3 min at 95 °C, and 40 cycles for 15 s at 95 °C, 30 s at 60 °C, and 30 s at 72 °C. Reaction specificity was confirmed through generation of a melting curve. Relative expression levels of silkworm SLC6 genes were calculated according to the $2^{-\Delta \Delta C_{t}}$ method. Statistical analyses were performed using Graphpad Prism version 5. Each sample was repeated in triplicate and all data are presented as the mean ± standard deviation (SD) with *n* = 3. A student’s *t*-test was used to evaluate statistical significance (* *p* < 0.05 and ** *p* < 0.01).

### 4.5. Starvation Experiment and JHA (Juvenile Hormone Analog) Treatment

To explore the potential function of SLC6 family genes that were highly and specifically expressed in the silkworm midgut, the starvation experiment was introduced. Newly molted larvae on the fifth instar were divided into 3 groups including feeding, starvation, and re-feeding. The feeding group (control group) was reared on fresh mulberry under the aforementioned conditions. The starvation group was subjected to the same conditions but without any diet. The larvae from the last group were starved for 24 or 36 h, and then fed with fresh mulberry for an extra 24 and 36 h, respectively. Midgut samples were collected from the three groups at 6, 24, 48, and 72 h and placed on ice. The samples were frozen in liquid nitrogen and stored at the −80 °C for total RNA isolation.

To determine the impact of hormones on mRNA transcript expression of silkworm SLC6 genes, the JHA treatment experiment was performed. Midguts were dissected derive from fourth molting larvae. The isolated midguts were cultured in Grace’s medium supplemented with 10% fetal bovine serum (FBS), 1% penicillin/streptomycin and JHA (1 μM) in the optimal atmosphere. Midguts incubated in culture with the same volume of acetone were used as controls. Samples from each group were collected in triplicate at 6, 12, and 18 h, for total RNA extraction.

## Figures and Tables

**Table 1:** Basic information of silkworm the solute carrier 6 (SLC6) family members.

SilkDB ID	Gene Name	Scaffold	Chr	Length (aa)	Exon	GenBank Accession Number	SilkDB Probe
BGIBMGA004216	*BmB(0)AT3-1*	nscaf2780	20	704	14	XP_004927289.1	sw06236
BGIBMGA004312	*BmOctT*	nscaf2789	20	706	19	XP_012545126.1	sw19499
BGIBMGA004570	*BmGAT1*	nscaf2800	27	602	12	XP_004923491.1	sw07510
BGIBMGA006164	*BmGT2*	nscaf2847	4	560	12	XP_004929221.1	sw03144
BGIBMGA006618-9	*BmDAT*	nscaf2855	10	619	UN	NP_001037362.1	sw00946 + sw06578
BGIBMGA006857	*BmINE*	nscaf2859	10	727	9	XP_004924838.1	sw12282
BGIBMGA007228	*BmNAT1*	nscaf2874	UN	640	14	XP_004927040.1	sw19553
BGIBMGA007228	*BmNAT2*	nscaf2874	UN	625	14	NP_001124343.1	sw19553
BGIBMGA008026-7	*BmGT1-L*	nscaf2889	9	635	12	XP_004922886.1	sw19800
BGIBMGA000927	*BmGT1*	nscaf1898	13	647	13	XP_012547426.1	sw08765
BGIBMGA010528	*BmSLC6-14*	nscaf2993	12	663	10	XP_004929448.1	sw14009
BGIBMGA011081	*BmSLC6-16*	nscaf3015	23	1145	15	XP_004921736.1	sw18195
BGIBMGA011849	*BmCT1-L*	nscaf3031	11	538	13	XP_012550400.1	sw16320
BGIBMGA011949-50	*BmB(0)AT3-2*	nscaf3032	11	724	5	XP_004924205.1	sw11049 + sw05356
BGIBMGA012340	*BmSLC6-15*	nscaf3040	1	637	10	XP_012551814.1	sw10873
BGIBMGA014231	*BmSERT*	nscaf98	8	596	13	NP_001037436.1	sw05387

B(0)AT3: broad substrate spectrum-neutral amino acid transporter 3; OctT: octopamine transporter; GAT: GABA transporter; GT: glycine transporter; DAT: dopamine transporter; INE: inebriated gene; NAT: nutrient amino acid transporter; GT1-L: glycine transporter 1-like; CT1: creatine transporter; SERT: serotonin transporter; SLC6: solute carrier 6; Chr: chromosome; UN: unknown; SilkDB: Silkworm Genome Database.

## Supplementary Materials

Supplementary materials can be found at www.mdpi.com/1422-0067/17/10/1675/s1.
